# The role of manganese over‐exposure in glutamate dysregulation and EEG outcomes in a mouse model of Alzheimer’s disease

**DOI:** 10.1002/alz.093434

**Published:** 2025-01-03

**Authors:** Rebecca A. Buchanan, Fiona E. Harrison

**Affiliations:** ^1^ Vanderbilt University, Nashville, TN USA; ^2^ Vanderbilt Brain Institute, Nashville, TN USA; ^3^ Vanderbilt University Medical Center, Nashville, TN USA; ^4^ Vanderbilt Brain Institute, Vanderbilt University, Nashville, TN USA; ^5^ Vanderbilt Memory & Alzheimer’s Center, Vanderbilt University Medical Center, Nashville, TN USA

## Abstract

**Background:**

Manganese (Mn) is an essential metal that serves as a cofactor for metalloenzymes important in moderating the glutamate/glutamine cycle and other oxidative stress pathways. Typically, Mn is acquired through the diet, however, Mn overexposure can arise through drinking inadequately treated well water or inhalation of Mn‐containing industrial byproducts. Mn toxicity disrupts dopaminergic neurotransmission resulting in a Parkinsonian disorder referred to as manganism. More recently, increased focus has been given to the role Mn toxicity in the progression of other neurodegenerative diseases including Alzheimer’s Disease (AD). Excess Mn may contribute to dysregulation of astrocytic glutamatergic transport, leading to elevated extracellular glutamate, excitotoxicity, and ultimately cell death, which are all key features of early AD pathology. We hypothesized that Mn toxicity drives glutamatergic signaling dysfunction to exacerbate pathology seen in AD including excitotoxicity and epileptiform activity.

**Method:**

APP^swe^/PSEN1^dE9^ mice, a mouse model of AD, and wild‐type littermates of both sexes were exposed to 50mg/kg MnCl_2_·4H_2_O or the vehicle subcutaneously three times over the course of a week as an acute high dose exposure of Mn. Increased seizure susceptibility in response to manganese and kainic acid (kainate receptor agonist) exposure was analyzed through surface cortical electroencephalogram recordings. Changes in glutamate reuptake transporter protein and mRNA expression from cortical and hippocampal tissue were quantified using western blot and qPCR.

**Result:**

APP^swe^/PSEN1^dE9^ mice have more cortical spiking activity over the course of exposure compared to WT mice. Effects of kainic acid were quantified over four hours and we found that Mn‐exposed mice have significantly decreased spiking over the first hour compared to vehicle treated mice. However, spiking activity increased over time in Mn‐treated mice, while spiking activity decreased in vehicle treated mice resulting in a significant reaction between treatment and time. There was no difference in spiking at the end of the four‐hour observation period.

**Conclusion:**

Dynamic responses to kainic acid and changes in glutamate reuptake transporter expression are consistent with glutamatergic changes that can contribute to excitotoxicity. Taken together, these findings support the hypothesis that manganese acutely interferes with glutamate functioning and could exacerbate phenotypes observed in AD.

